# Supplementary material for: Safe-Shields: Basal and Anti-UV Protection of Human Keratinocytes by Redox-Active Cerium Oxide Nanoparticles Prevents UVB-Induced Mutagenesis
Source: Antioxidants (Basel). 2023 Mar 20;12(3):757. doi: 10.3390/antiox12030757 (PMC10045349; doi:10.3390/antiox12030757)
Supplement: Supplementary file 1 [file antioxidants-12-00757-s001.zip › antioxidants-2283846-supplementary.pdf]

antioxidants

Supplementary Materials

Supplementary Table S1. Summary of CNP powder characteristics: nanoparticles size was measured by SEM, specific surface area (SSA) by BET, mean hydrodynamic diameter (d) and Zeta potential (Z pot) in deionized water by Zetasizer.

Crystal structure	Size (nm)	SSA (m^2/g)	d (nm)	Z pot (mV)
Fluorite	9 ± 2	74	164 ± 8	-23 ± 1

Supplementary Table S2. Summary of TNP powder characteristics: nanoparticles size was measured by SEM, specific surface area (SSA) by BET, mean hydrodynamic diameter (d) and Zeta potential (Z pot) in deionized water by Zetasizer.

Crystal structure	Size (nm)	SSA (m^2/g)	d (nm)	Z pot (mV)
Anatase	10 ± 2	50	228 ± 4	-35 ± 1
